# Polycarbonate-Based Blends for Optical Non-linear Applications

**DOI:** 10.1186/s11671-016-1256-5

**Published:** 2016-02-13

**Authors:** F. Stanculescu, A. Stanculescu

**Affiliations:** Faculty of Physics, University of Bucharest, 405 Atomistilor Street, P.O. Box MG-11, Bucharest-Magurele, 077125 Romania; National Institute of Materials Physics, 105 bis Atomistilor Street, P.O. Box MG-7, Bucharest-Magurele, 077125 Romania

**Keywords:** Organic/organic composite film, Polymeric matrix, Amidic compounds, Optical properties, SHG, 42.70.Jk, 68.60.-p, 78.66.-w

## Abstract

This paper presents some investigations on the optical and morphological properties of the polymer (matrix):monomer (inclusion) composite materials obtained from blends of bisphenol A polycarbonate and amidic monomers. For the preparation of the composite films, we have selected monomers characterised by a maleamic acid structure and synthesised them starting from maleic anhydride and aniline derivatives with –COOH, –NO_2_, –N(C_2_H_5_)_2_ functional groups attached to the benzene ring. The composite films have been deposited by spin coating using a mixture of two solutions, one containing the matrix and the other the inclusion, both components of the composite system being dissolved in the same solvent. The optical transmission and photoluminescence properties of the composite films have been investigated in correlation with the morphology of the films. The scanning electron microscopy and atomic force microscopy have revealed a non-uniform morphology characterised by the development of two distinct phases. We have also investigated the generation of some optical non-linear (ONL) phenomena in these composite systems. The composite films containing as inclusions monomers characterised by the presence of one –COOH or two –NO_2_ substituent groups to the aromatic nucleus have shown the most intense second-harmonic generation (SHG). The second-order optical non-linear coefficients have been evaluated for these films, and the effect of the laser power on the ONL behaviour of these materials has also been emphasised.

## Background

The class of organic materials is promising for applications in the processing of information, telecommunication and integrated optics. The organic compounds characterised by theoretical high non-linearities and rapid electro-optic responses represent an important alternative to the inorganic non-linear materials for second-harmonic generation (SHG), frequency mixing, optical parametric oscillation, optical bistability and electro-optic modulation. These materials could be considered as the optical materials of the future because their molecular nature combined with the versatility of the synthetic chemistry offer ways to modify and optimise the molecular structure with the purpose to increase the non-linear response [1, 2].

Because of their unique chemical structure conferred by the π bonds, the organic molecules show the highest non-resonant optical non-linearities with a fast response time which is limited only by the laser pulse. By comparison, the resonant optical non-linearities have a response time determined by the lifetime of the excitation. The values of the second-order susceptibility in these materials are determined by the packing density of the optical non-linear entities in a macroscopic structure. Most of the organic materials crystallise in centrosymmetric structures, and for this reason, it is difficult to generate significant second-order optical non-linear effects. In addition, the preparation and processing of the bulk organic materials, like the organic crystals, are complicated processes involving a lot of time [3–5]. Anyway, during the crystal growth, non-centrosymmetric structures could be incorporated as dopant in the bulk material. Thus, the organic crystals can be used as crystalline “host” matrices for organic or inorganic “guests” [6–11].

For the generation of the optical non-linear (ONL) phenomena, we must consider some specific criteria in the selection of the functional groups substituted to the benzene nucleus of the organic compounds, such as the extension of the π-electron system, length of the conjugated chain and planarity of the molecule [2]. For example, a great interest is paid to the study of the π-conjugated polymeric structures associated with high values of the high order polarizability [1, 2].

Different types of polymers have been synthesised until now showing optical, electrical, chemical, mechanical and thermal properties adequate for a large range of applications from daily life facilities to high-performance devices for the aerospace industry and medicine. The properties of these materials can be anticipated and tailored by molecular design, controlled synthesis and processing making them useful for photovoltaics, batteries, membranes and composite and as smart materials for coatings, sensors and biomimicry.

Polymers also focus the interest because the properties at the nanoscale dimension, in the form of polymeric nanoparticles, recommend them for a variety of applications such as toners, coatings, adhesives, instrument calibration standards and column-packing materials for chromatography, biomedicine and biochemical analysis [12]. In addition, the polymer-based nanoscale structures, realised using a variety of techniques including the atomic force microscopy [13, 14], laser-assisted nanoimprint lithography [15] and generation of polymeric nanofibers by electrospinning [16], are also promising for many applications from bioengineering to optics and from self-cleaning surfaces to sensors. For example, single-walled carbon nanotube-enriched polymer composite materials have been used for a flexible, enhanced sensibility X-ray detector [17].

In the last years, a special interest has been paid to the field of composite materials combining the properties of an inorganic vitreous or organic polymeric matrix with those of inorganic or organic inclusions [18–24]. Significant research has been devoted to the investigation of the self-assembling of different types of nanoparticles in a polymeric-ordered matrix [25]. In the case of organic inclusions, the main attention was focused on the identification and preparation of some molecular structures containing functional groups adequate for the generation of optical non-linear phenomena [26–31].

The polymeric matrices are preferred to the inorganic vitreous ones because, on the one hand, their preparation does not impose restrictions (they are soluble in different ordinary solvents and involve low processing temperatures), and on the other hand, polymers show adequate optical properties (large range of transparency and high, homogeneous refraction index). The extension of the π-electron delocalisation from one repeating polymer unit to another which depends on the details of the polymer structure determines the properties of the polymer, including the optical non-linearities and sometimes the increased electrical conductivity [1]. Thus, the conductor polymers become interesting for non-linear optics, but a direct connection between these two properties was not found yet. Another advantage of the polymeric materials is their easy processability favouring the preparation of thin films on different substrates.

The incorporation of organic inclusions can be realised using a variety of methods leading to different types of organic:organic composites showing second-order optical non-linearities: (1) by adding the active components into a polymeric matrix and generating a “host:guest” system, (2) by covalently bonding the active component to the chain of the polymer as side chains or (3) by incorporating the active component inside the main chain of the polymer [2]. The side-chain incorporation of the active molecules is sometimes preferred because the reduced solubility of the “guest” molecules into the “host” matrix, in the “host:guest” system, allows only a low or medium level of inclusion incorporation. Thus is limited the concentration of optical non-linear entities with an effect on the intensity of the ONL phenomena.

The preparation of an organic:organic composite by the incorporation of optical non-linear “guest” organic molecules in a “host” polymeric matrix is much easier than the preparation of an inorganic:inorganic composite. The advantage of the “host:guest” system is the simplicity and reproducibility that is better than that obtained using the other methods of incorporation presented above.

This paper presents the study of an organic:organic composite material obtained from a blend of polycarbonate of bisphenol A with an amidic monomer, characterised by significant optical non-linear properties.

The ONL materials require chemical and thermal stability and low optical loss at the operating wavelength [32]. Polycarbonate of bisphenol A is a polymer which has been intensively studied and is a good candidate for electro-optic “host” because it shows good transparency and processability and good thermal properties (glass transition temperature 145 °C, melting temperature = 155 °C [33, 34]) and thermo-mechanical stability [32, 35, 36]. The amidic derivatives are thermally stable, starting to degrade over 200 °C [37]. When introduced in polycarbonate, they can also determine the stabilization of polymer against the degradation generated by UV light through photo-oxidation processes [38]. We obtained a material combining the good optical, thermal and film-forming properties of polycarbonate with the good optical non-linear properties and thermal stability of monomer.

The first step in the preparation of the proposed “host:guest” system was to prepare the amidic monomeric structures with active functional groups substituted to the aromatic ring. The main experimental problem associated with the proposed organic:organic “host:guest” system is the incorporation of the maleimidic monomers in the polymeric matrix, because they show processing limitations determined by the solubility and fusibility properties [39–43].

These monomers were obtained by attaching lateral chains with different lengths to the main chain of HOOC–HC=CH–CO. These lateral chains contain single-ring aromatic derivative mono substituted with one of the following groups, –COOH or –NO_2_ or –N(C_2_H_5_)_2_, or single-ring aromatic derivative double substituted with two –NO_2_ groups. These lateral chains are attached to the main chain by intercalated groups of –NH– or –NH–NH–.

The properties of these polymer:monomer films prepared from solution by spin coating have been investigated using spectrophotometric (ultraviolet-visible (UV-Vis) transmission and photoluminescence) and microscopic (scanning electron microscopy (SEM) and atomic force microscopy (AFM)) methods. Some of these composite films have shown significant optical non-linear effects.

## Methods

**Fig. 1:**
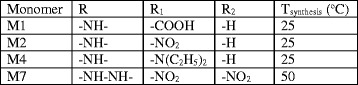
Monomers synthesis schema where *a* = maleic anhydride, *b* = aniline derivatives, *c* = synthesised monomers (M1; M2; M4; M7) characterised by the following chemical structures:

We have synthesised the monomers, the “guest” component in the composite system, from maleic anhydride and some aniline derivatives. These compounds are characterised by a maleamic acid structure with different substituent groups on the aromatic ring (Fig. 1) such as the following: R (–NH– or –NH–NH–); R_1_ (–COOH or –NO_2_ or –N(C_2_H_5_)_2_); R_2_ (–NO_2_).

We have used as starting material the maleic anhydride (MA) (Fluka, 95 % purity) purified by recrystallization in chloroform. The reaction partners were para-aminobenzoic acid, para-nitroaniline, 4,*N*,*N* diethyl aniline and 2,4 dinitrophenylhydrazine (all supplied by Fluka and having analytical purity) and have been utilised without supplementary purifications.

The synthesis of the maleamic acids involves the opening of the anhydride cycle (Fig. 1). We have slowly added the solution of MA in dimethylformamide (DMF) to the solution of aniline derivative in DMF. The final solution is easily stirred at room temperature for 2 h. The compounds are obtained by semiamide (maleamic acid) precipitation in water with ice, filtration and recrystallization from methanol with different final yields depending which aniline derivative has been used [37]: 17 % for the monomer synthesised with para-aminobenzoic acid (M1), 34 % for the monomer synthesised with para-nitroaniline (M2), 27 % for the monomer synthesised with 4,*N*,*N* diethyl aniline (M4) and 80 % for the monomer synthesised with 2,4 dinitrophenylhydrazine (M7). The synthesised compounds, M1, M2, M4 and M7, are characterised by high solubility in DMF and high melting points situated between 200 and 230 °C [37], in concordance with the values presented in the literature for the maleimidic compounds.

The next stage, preparation of the composite films, implies two important steps: (1) preparation of the “mother solution” of the polymer matrix and monomer inclusion and (2) deposition of the films by spin coating. A common procedure for the preparation of polymer:monomer blends is the dissolution of polymer and monomers in appropriate solvents followed by the mixing of these solutions. The solvent favours the pushing apart of the polymer chain and functional groups of the monomer allowing stronger or more extensive intercomponent interactions. The behaviour of the blend is strongly influenced by the nature and polarity of the solvent, chemical structure and molecular mass of the components. Therefore, a very important step in the preparation of the “mother solution” was the selection of the solvent.

We have tested different solvents such as benzene, ethylene glycol and DMF. The solubility of the monomer and the evaporation rate of the solvent are crucial in this selection, because they affect the process of film preparation. DMF has been selected for the deposition of the composite layers from solution by spin coating because both monomers and polymer show a good solubility in DMF and because DMF shows a relatively good volatility at room temperature.

The polymer, polycarbonate of bisphenol A, and the selected monomer are separately dissolved in DMF, and after that, these two solutions are mixed. We have obtained the “mother solutions” with a polymer concentration of 0.06 g/cm^3^ and monomer (M1; M2; M4; M7) concentration of 0.03 g/cm^3^. These mixtures will be used for the deposition of the films by spin coating on glass and etched silicon wafers utilizing a Chemat Technology KW-4 A equipment. The deposition substrates have been previously cleaned in acetone, alcohol ethylic and deionised water. All the films have been prepared in the same experimental conditions. The spinning time, *t*, and the spinning speed, *v*, for every stage of the deposition process were kept constant: for the spreading stage (*t*_1_ = 6 s, *v*_1_ = 700 rpm) for the homogenization stage (*t*_2_ = 10 s, *v*_2_ = 620 rpm).

The UV-Vis transmission and photoluminescence (PL) spectra of the polycarbonate:monomer composite films have been obtained with a Spectrophotometer Carry 5000 and Spectrofluorimeter Edinburgh Instruments F-900, respectively. Fourier transform infrared (FTIR) spectra have been drawn with a Perkin Elmer BX Spectrophotometer offering details about the layer composition. The morphology of the layers was investigated by SEM performed with a Hitachi S 3400 apparatus at an acceleration voltage *V*_acc_ = 20 kV and two magnifications for each sample. The samples have been coated prior to analysis with a gold layer deposited by sputtering to prevent charging. The surface topography of the films has been evidenced by AFM realised with MultiView 4000 Nanonics System working in tapping mode using a probe with a diameter of 10 nm, resonance frequency around 35 kHz, factor of merit of 1700 and an investigation area of 10 μm × 10 μm. We have obtained details about the roughness of the films evaluating the surface amplitude parameters, root mean square (RMS) and roughness average (RA).

Qualitative information about the structure of the layers has been obtained by X-ray diffraction measurements realised with a Bruker D8 Advance Diffractometer at the grazing incidence, using the Cu K_α1_ line, an acceleration voltage of 40 kV and an anodic current of 40 mA.

The optical non:linear (ONL) properties of the prepared organic:organic composite films were investigated with an experimental set-up including a femtosecond pulse laser. For the second-harmonic excitation, we have used an ultrashort pulsed laser Spectra Physics “Tsunami” with a maximum emission wavelength of 800 nm, pulse duration of 60 fs, frequency of 80 MHz and power varying between 10 and 550 mW. The laser beam was focused on the sample surface using a high N.A. Mitutoyo microscope objective. The sample was mounted on a motorised Thorlabs XYZ stage. Details about this configuration have been presented in a previous paper [37].

## Results and discussion

**Fig. 2:**
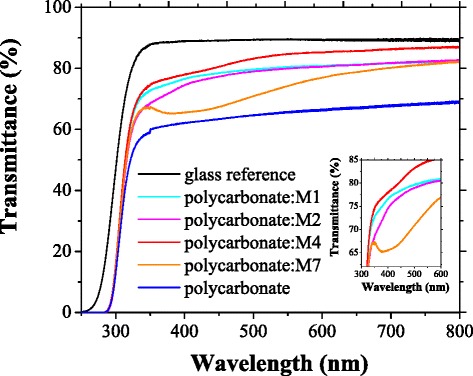
Transmission spectra of polycarbonate and composite films of polycarbonate:M1, polycarbonate:M2, polycarbonate:M4 and polycarbonate:M7 deposited by spin coating on glass substrate. Details about the shape of the absorption edge of the mentioned composite films are presented in *inset*

All the prepared films have shown a good transmission in a large spectral range from UV to NIR, between 350 and 800 nm (Fig. 2).

The UV-Vis transmission spectra of the films of polycarbonate and polycarbonate with monomer inclusions show a steep fundamental absorption edge which is slightly shifted to a longer wavelength compared to the fundamental absorption edge of the glass substrate (Fig. 2). A slight tendency towards the structuring of the absorption edge and the appearance of two absorption steps situated around 300-350 nm and over 380 nm (Fig. 2) were evidenced in some composite films. This structure of the absorption edge can be explained by the presence in the amidic monomer structure of the carbonyl groups (Fig. 1) that contain lone electron pairs of O atoms, designed non-bonding electrons. Such an electron could be promoted to an unoccupied π orbital giving the (n, π*) excited state instead of the (π, π*) state [44]. The carbonyl groups of different monomer molecules forming the inclusion phase could be situated close one to another showing strong interaction between their electrons and favouring the splitting of the carbonyl (n, π*) level into two bands causing the appearance of the two absorption steps [44]. The same effect could appear in monomers based on a nitrous aromatic derivative because the nitrous group (Fig. 1) also has lone electron pairs of O atoms and its behaviour is similar to that of the carbonyl group. At ~330 nm, the lone electron pairs transition is overlapped with the π-π* transition of the benzene rings in the polymeric matrix and monomeric inclusion.

The possibility to generate a strong interaction between molecules and to produce the splitting of the energetic level at the low concentration of monomer used in our experiments (0.03 g/cm^3^) is lower for the monomer with a small molecule and substituent group with a shorter chain (M2 and M1) than for a monomer with a large molecule and substituent/intercalated groups with a longer chain (M4 and M7). Therefore, the absorption broad peak situated at 380–450 nm is attenuated and practically disappears (Fig. 2) in the composite material containing a monomer with a shorter chain substituent group (M1 and M2). With the increase in the conjugation length in monomers with a longer chain of the substituent and intercalated functional groups (M4 and M7), the two-step structure, determined by the (n, π*) splitting [44] mentioned above, develops. The spectrum shows a step situated between 300 and 350 nm and a shoulder situated between 380 and 450 nm (Fig. 2). The shape of the absorption edge in M7 with a strong shoulder between 380 and 450 nm suggests a specific arrangement characterised by a close packing of the molecules in the solid state. In this arrangement, some carbonyl/nitrous groups from different molecules are brought close to each other favouring the splitting of the energetic levels. Therefore, the structured absorption edge evidenced by UV-Vis transmission spectra is a consequence of both the organic molecular structure and solid-state packing of molecules.

The shape of the transmission curves for samples containing inclusions of monomers M2, M4 and M7 is associated with a certain degree of light scattering on the compositional non-homogeneities of the layers. The spin-coating method used to prepare the polycarbonate:monomer composite layers does not assure a rigorous control of the homogeneity of the layers. The light scattering takes place on the monomer inclusions (aggregates) with different dimensions and is more significant in polycarbonate:M7 compared to the other composite layers (Fig. 2).

It is difficult to prepare uniform and homogeneous films by spin coating, especially from the solution containing two different components dissolved in solvent. The pre-wetting of the substrate with a few drops of the same solvent used to prepare the “mother solution”, DMF, offered a way to improve the spreading of the “mother solution” over the substrate surface and to reduce the generation of the “finger”-type instabilities. This is realised by controlling the forces acting between the substrate and the “mother solution” by the forces acting only between the substrate and the solvent molecules deposited during the pre-wetting stage.

**Fig. 3:**
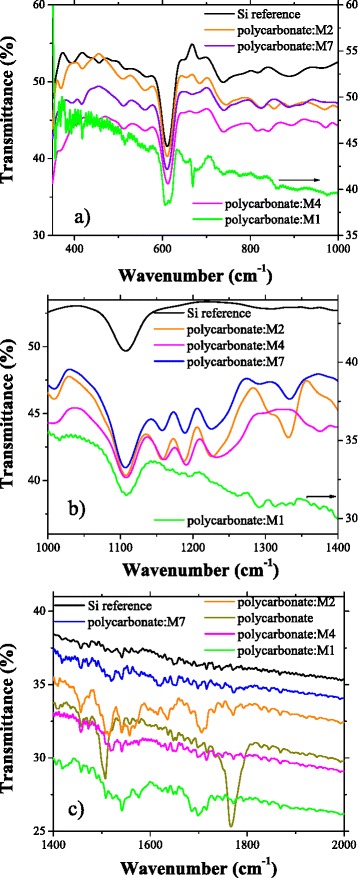
FTIR spectra of polycarbonate and composite films of polycarbonate:M1, polycarbonate:M2, polycarbonate:M4 and polycarbonate:M7 deposited by spin coating on Si substrate in different spectral range: 370–1000 cm^−1^ (**a**), 1000–1400 cm^−1^ (**b**) and 1400–2000 cm^−1^ (**c**)

The FTIR spectra gave us qualitative information about the composition of the prepared organic composite layers. The characteristic bands of monomers [45] have been found in the composite films deposited on silicon wafers (Fig. 3a–c): 1537 and 1505 cm^−1^ for the amidic group (N–H), 1623 cm^−1^ for the carboxylic and amidic group, 1294 cm^−1^ for the carbonyl group, 848 cm^−1^ for double bound (CH=CH), and 675 cm^−1^ for single bond (C–H). These absorption peaks could be shifted and/or widened under the influence of the polymeric matrix. The presence of a maleamic acid structure associated with the monomer is evidenced in ll these spectra by the disappearance of the MA carbonyl group band situated at 1855 cm^−1^ and the weakening until disappearance of the peak situated at 1774 cm^−1^ [43, 46, 47]. The weakening/disappearance of the last mentioned peak confirms that it is possible to simultaneously have amidic and imidic monomer structures in the polymeric matrix.

**Fig. 4:**
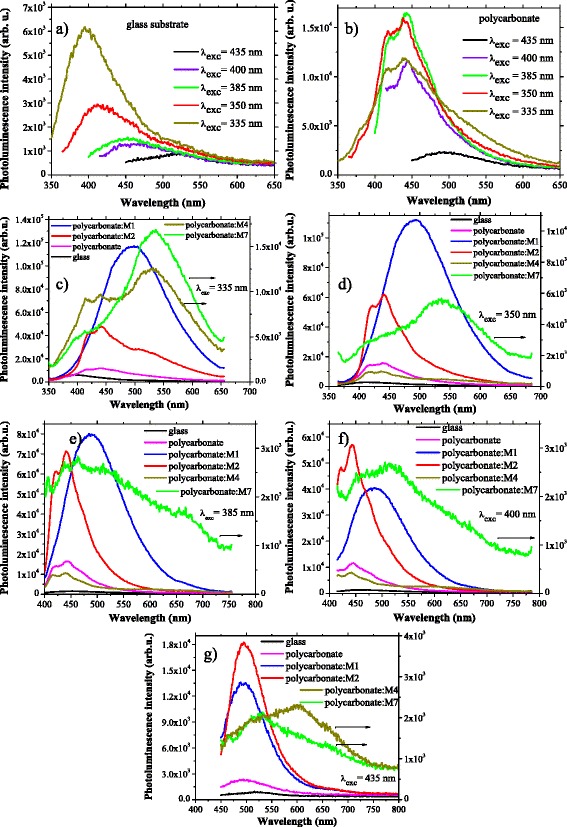
PL spectra of glass substrate at different excitation wavelengths (**a**); PL spectra of polycarbonate film deposited by spin coating on glass substrate at different excitation wavelengths (**b**); comparative PL spectra of polycarbonate:M1, polycarbonate:M2, polycarbonate:M4 and polycarbonate:M7 composite films deposited by spin coating on glass substrate at a given excitation wavelength: *λ*_exc_ = 335 nm (**c**), *λ*_exc_ = 350 nm (**d**), *λ*_exc_ = 385 nm (**e**), *λ*_exc_ = 400 nm (**f**) and *λ*_exc_ = 435 nm (**g**)

The photoluminescence of the glass substrate is more significant at excitation with UV wavelengths and the emission peak slightly red shifts from 400 nm to longer wavelengths, 525 nm, when the excitation wavelength increases from 335 to 435 nm (Fig. 4a). The film of polycarbonate deposited on glass shows at excitation with wavelengths of 335, 350 and 385 nm a split emission band with two maxima situated at 415 and 435 nm. At excitation with wavelengths longer than 400 nm, the emission is dominated by the emission properties of the glass substrate (Fig. 4b).

We have remarked different emission properties of the composite films deposited from the mentioned blends, at the excitation with the selected excitation wavelengths situated between 335 and 435 nm: 335 nm (Fig. 4c), 350 nm (Fig. 4d), 385 nm (Fig. 4e), 400 nm (Fig. 4f) and 435 nm (Fig. 4g). The layers of polycarbonate:M1 and polycarbonate:M2 showed stronger emission compared to polycarbonate:M4 and polycarbonate:M7. The polycarbonate:M1 film shows a strong emission peak with a maximum situated around 500 nm, independently of the excitation wavelength. The polycarbonate:M4 and polycarbonate:M7 films are characterised by weaker well-defined peaks with a maximum around 550 nm only for *λ*_exc_ = 335 nm (Fig. 4c). The peak maintains its position, 550 nm, also for excitation of the polycarbonate:M7 film with a wavelength of 350 nm (Fig. 4d). A large emission band is evidenced for the polycarbonate:M7 film at excitation with wavelengths of 385, 400 and 435 nm and for polycarbonate:M4 film at excitation with a wavelength of 435 nm. We have evidenced for polycarbonate:M2 film at excitation with wavelengths of 335, 350, 385 and 400 nm a strong emission band situated between 400 and 500 nm with two maxima centred at 420 and 450 nm which became at excitation with a wavelength longer than 400 nm a single maximum emission band centred at 500 nm. This behaviour is in concordance with the emission characteristics of the films of monomers with maleamidic acid structure deposited by matrix-assisted pulsed laser evaporation, which have been previously presented [48]. This confirms the preservation of the emission properties of the monomers by embedding in a polycarbonate matrix.

The transmission and photoluminescence properties are important because they are affecting all other optical properties of the films, including the generation of optical non-linear phenomena. For example, it is expected a substantially reduced loss in the second-harmonic signal for materials with good transparency and low photoluminescence at the wavelength corresponding to the second harmonic.

Other important parameters affecting the properties of the films are the morphology and structure, and they depend on the particularities of the molecular chemical structure of the monomer inclusions and polymeric matrix. The degree of order in the polycarbonate of bisphenol A:MX (*X* = 1,2,4,7) composite films has been qualitatively evaluated by X-ray diffraction measurements, which have revealed the lack of long range order in the spin-coated films. The film is mostly amorphous, and the molecules of monomer are randomly oriented because we have not identified any reflection peaks on the X-ray diffractograms. The general shape of the diffraction pattern is determined mostly by the properties of the glass substrate.

**Fig. 5:**
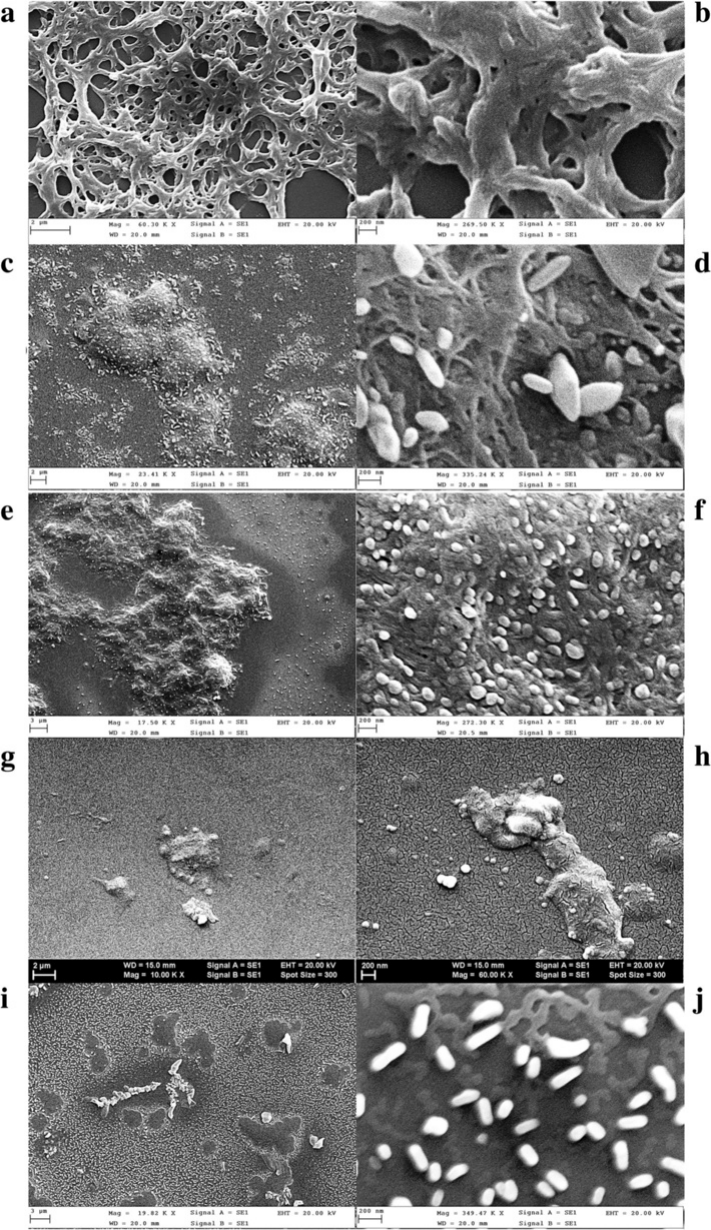
SEM images at two different magnifications of the films deposited by spin coating on glass: polycarbonate matrix (**a**, **b**), polycarbonate:M1 (**c**, **d**), polycarbonate:M2 (**e**, **f**), polycarbonate:M4 (**g**, **h**), polycarbonate:M7 (**i**, **j**)

The morphology of the films depends on the interaction between substrate and polymer, substrate and monomer, and substrate and solvent. The silicate glass surface has a hydrophilic character, and the interaction with DMF is strong. The polymeric chains tend to wet the glass surface because the interactions between the polycarbonate of bisphenol A and the substrate are stronger than the interactions between the solvent molecules and the substrate. After, the evaporation of the solvent remains a specific solidification structure of the polymer, corresponding to the lowest free energy of the system [49, 50]. This structure is characterised by the overlapping of 2D networks which delimits free spaces (Fig. 5a, b) of different dimensions.

The concentration of polycarbonate in DMF and the physical parameters of the deposition process, such as the spinning speed and duration, are the same for all the prepared samples, and therefore, the observed morphology is mainly the result of the properties of the monomers. SEM images confirm the existence of two different phases in the films deposited by spin coating on glass: the polymeric matrix phase containing the dispersed phase of monomer inclusions. The film is continuous but non-uniform (Fig. 5c–j) with inclusions (aggregates) with sizes between hundreds of nanometers (Fig. 5d, f, h, j) and few micrometres (Fig. 5c, e, g, i) which are randomly distributed in the polymeric matrix. The SEM images of polycarbonate:M1 and polycarbonate:M4 composite films show beside the large dimension inclusions, a significant density of inclusions of smaller dimension, <100 nm (Fig. 5d, h).

The above-mentioned morphology is determined by the monomer molecules showing polarity which is determined by the difference in the electronegativity of the group substituent on the aromatic nucleus. The interaction between the polar molecule of monomer and substrate is strong and comparable with the interaction between polymer and substrate. The process of monomer molecule aggregation to generate inclusions in the polymeric matrix can be favoured by the rapid solvent evaporation and strong polycarbonate-monomer-repulsive interaction. The polycarbonate covers the substrate, and the monomer aggregates as a distinct dispersed phase [51].

**Fig. 6:**
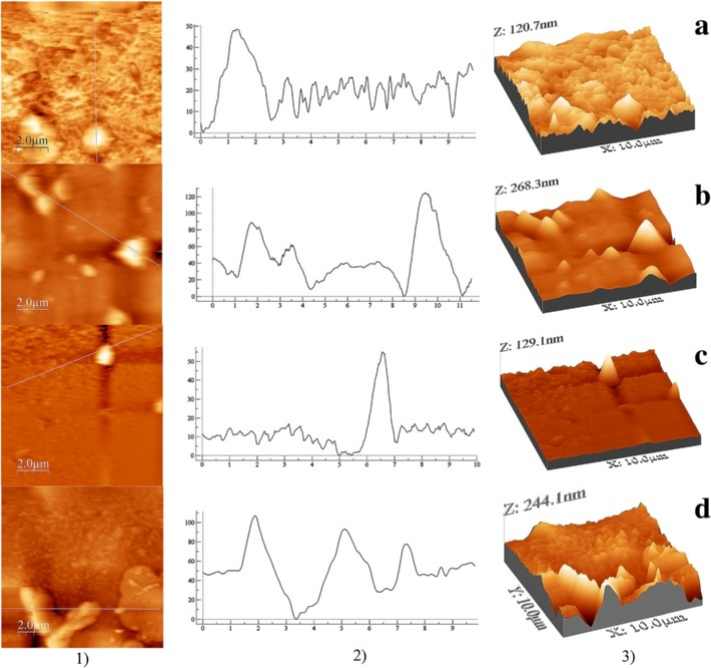
2D AFM images (**1**), profile line where OX represents position in micrometres and OY represents height in nanometres (**2**) and 3D AFM images (**3**) of the composite films deposited by spin coating on glass: polycarbonate:M1 (**a**), polycarbonate:M2 (**b**), polycarbonate:M4 (**c**) and polycarbonate:M7 (**d**)

Details about the surface topography of the spin-coated films have been obtained by processing the AFM images (Fig. 6) with WSx4.0 software to evaluate the parameters RMS and RA. The film of polycarbonate of bisphenol A deposited on glass substrate shows a low roughness (RMS = 3.2 nm; RA = 1.7 nm) comparable with the roughness of the glass substrate (RMS = 4.0 nm; RA = 1.9 nm). The highest roughness has been obtained for the films of polycarbonate:M7 (RMS = 29 nm; RA = 20 nm) and polycarbonate:M2 (RMS = 25 nm; RA = 17 nm). This roughness can be correlated with the presence of inclusions having a mostly large dimension. As already emphasised above, the distinct phase represented by inclusions is also involved in the light scattering which affects the shape of the transmission spectra of the prepared composite films (Fig. 2). A lower roughness was obtained for polycarbonate:M1 (RMS = 13 nm; RA = 10 nm) and polycarbonate:M4 (RMS = 7.2 nm; RA = 3.4 nm) and is correlated with the morphology characterised by the presence of a mixture of small and large dimension inclusions spread in the matrix of polycarbonate, already revealed by SEM (Fig. 5d, h).

An estimation of the dimension of the inclusions for each deposited film has been realised from the profile line obtained with WSxM4.0 software. The largest width of the inclusions was around few micrometres and the smallest <100 nm. Correlating the AFM results (Fig. 6) with SEM details (Fig. 5), the morphology of polycarbonate:M1 film (Fig. 6a) and polycarbonate:M4 film (Fig. 6c) is dominated by small dimension inclusions while the morphology of polycarbonate:M2 film (Fig. 6b) and polycarbonate:M7 film (Fig. 6d) is dominated by large dimension inclusions. The highest peak value of the inclusions associated with high “hills” giving a sharpened morphology has been evidenced in polycarbonate:M2 film and polycarbonate:M7 film (Fig. 6b, d). Therefore, polycarbonate:M2 and polycarbonate:M7 films are characterised by wider and higher inclusions corresponding to high roughness by comparison with polycarbonate:M1 and polycarbonate:M4 films characterised by narrower and lower inclusions corresponding to low roughness.

**Fig. 7:**
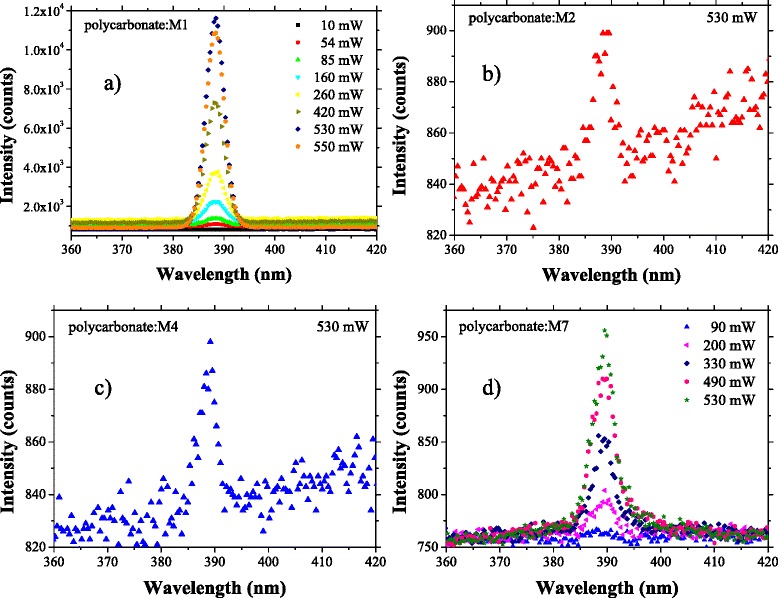
Optical non-linear phenomena (SHG) intensity versus wavelength for different average laser power in polycarbonate:M*X* (*X* = 1, 2, 4, 7) composite films deposited by spin coating on glass: polycarbonate:M1 (**a**), polycarbonate:M2 (**b**), polycarbonate:M4 (**c**) and polycarbonate:M7 (**d**). The glass substrate has no contribution to SHG

The optical non-linear properties of these composite films have also been investigated (Fig. 7). The strong second-harmonic (SH) intensity can be explained by the inclusions of monomers. The monomers are characterised by highly polarizable substituent groups (–NH–, –NH–NH–, –COOH, –NO_2_, –N(C_2_H_5_)_2_) which interact with the delocalised cloud of π electrons of the benzene nucleus generating strong inductive and mezomeric effects.

The absorption and photoluminescence properties of the composite films determine the intensity of the SH signal and the generation of other optical non-linear phenomena, like two-photon fluorescence emission (TPF).

The partial absorption of the second harmonic can determine the appearance of other optical non-linear effects, like TPF, because the absorption of two photons with *λ* = 800 nm of the laser beam is equivalent with the absorption of one photon with *λ* = 400 nm, the wavelength corresponding to SH emission. We have not evidenced any significant absorbance of the second-harmonic wavelength situated around 400 nm in the films deposited from the mentioned polymer:monomer blends (Fig. 2), and therefore, we have not evidenced the TPF.

The spin-coated layer of polycarbonate:M1 shows the strongest SHG (Fig. 7a) which is determined by the significant difference in electronegativity between the two groups attached to the aromatic ring: –NH– and –COOH. The intensity of the SH signal increases with the increase of the laser power from 10 to 550 mW. A significant SH signal is also given by the spin-coated layer of polycarbonate:M7 (Fig. 7d) while the spin-coated layers of polycarbonate:M2 (Fig. 7b) and polycarbonate:M4 (Fig. 7c) have presented very weak SH signals, which became more intense only at a laser power >500 mW.

**Fig. 8:**
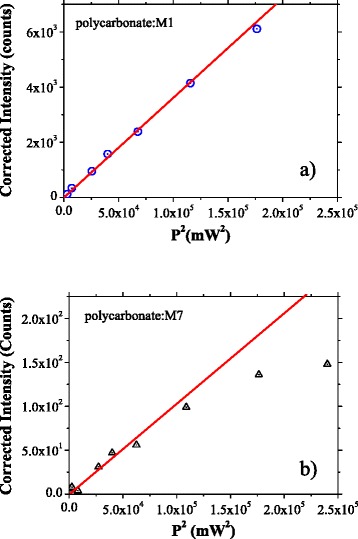
Dependence SH intensity versus square of laser average power for: polycarbonate:M1 (**a**) and polycarbonate:M7 (**b**). Corrected intensity means the intensity of SH signal minus the background measured in the same experimental conditions

A measure of the optical non-linear properties of the material is represented by the slope of the plot showing the intensity of SH versus the square of laser average power [52]. We have drawn these plots for the composite films polycarbonate:M1 (Fig. 8a) and polycarbonate:M7 (Fig. 8b) showing the strongest SHG.

The slope of these plots suggested a lower second-order ONL coefficient in the layer prepared from the blend polycarbonate:M7 compared to the layer prepared from the blend polycarbonate:M1. At a laser power higher than 380 mW, we have observed a saturation of the SH signal in the polycarbonate:M7 layer (Fig. 8b). This phenomenon can be associated with conformational changes of the molecules determined by the thermal effect of the laser beam and involve the reorientation of the dipoles and changes in the polarizability of the sample. In the sample polycarbonate:M1, we have not evidenced any saturation of the SH signal for laser power up to 480 mW (Fig. 8a).

Nevertheless, the SH intensity is lower in polycarbonate:M7 compared to polycarbonate:M1 (Fig. 7a, d), despite the low loss by PL emission at excitation with wavelength of 400 nm corresponding to the wavelength of the SH. This behaviour of the polycarbonate:M7 layer can be explained by the high roughness and morphology of this composite film determined by inclusions of large dimension randomly distributed, favouring both the bulk and surface scattering and affecting the transmission.

The main advantages of using these blends in ONL applications are related to the ONL properties of the aniline derivative inclusions and very good transparency of the polycarbonate matrix, to easy processing from the solution offering the possibility to obtain films on different large- area substrates including flexible substrates and to the possibility to tailor the properties of the blend by controlling the concentration of aniline derivatives in the polycarbonate matrix. In addition, the rigorous control of the deposition parameters can assure a more homogeneous distribution of these inclusions in the polymeric matrix. Therefore, a solution to finely modulate the ONL properties of the composite material is offered by appropriate changes in the composition of the polycarbonate:monomer system.

## Conclusions

Organic:organic blends have been used to prepare by spin coating films containing inclusions of amidic monomers in a matrix of polycarbonate of bisphenol A. The monomers synthesised from maleic anhydride and some aniline derivatives are characterised by a maleamic acid structure and contain –NH– or –NH–NH– intercalated groups and COOH or –NO_2_ or –N(C_2_H_5_)_2_ substituent groups on the aromatic nucleus. The FTIR spectra have evidenced the possibility of the simultaneous presence of the amidic and imidic molecular structures in the polymeric matrix.

The UV-Vis spectra have revealed the two-step absorption process, due to the presence of the lone electron pairs of the O atoms in the carbonyl and nitrous groups. These electrons are associated with the (n, π*) excited states whose splitting is favoured by the specific close packing of the molecule in the solid state. The second step of the absorption edge in the transmission curve of films of polymer with amidic monomer inclusions became stronger for monomers with structures characterised by extended conjugation, meaning groups with a longer chain attached to the benzene ring (M4 and M7).

At excitation with wavelengths around the wavelength of SH signal situated at 385, 400 and 435 nm, the composite films polycarbonate:M1 and polycarbonate:M2 have shown the most significant photoluminescence emission, while the composite films polycarbonate:M4 and polycarbonate:M7 have shown the weakest emission.

Correlation between SEM and AFM measurements have shown different morphologies of the film deposited by spin coating on glass substrates, characterised by inclusions with size between hundreds of nanometers and few micrometres. In polycarbonate:M1 and polycarbonate:M4, we have also identified smaller inclusions with dimension <100 nm. The morphology is determined by the strength and attractive/repulsive character of the interactions between the solvent, polymer, monomers on the one hand and the substrate for deposition on the other hand. The polycarbonate:M2 and polycarbonate:M7 films are characterised by wider and higher inclusions and show the highest roughness. On the contrary, the polycarbonate:M1 and polycarbonate:M4 films are characterised by narrower and lower inclusions and show the lowest roughness.

The strongest SHG effect has been evidenced in polycarbonate:M1 composite film. Monomer M1 contains highly polarizable substituent groups to aromatic ring, –HN– and –COOH, characterised by a high difference in electronegativity. These groups determine inductive/mezomeric effects by coupling with the polarisable aromatic nucleus. Despite its higher photoluminescence at excitation with wavelength around the SH wavelength compared to the other composite films, the polycarbonate:M1 film shows strong SHG. This behaviour can be explained by its very good transmission and low roughness.

## Abbreviations

AFM: atomic force microscopy
arb.u.: arbitrary units
DMF: dimethylformamide
FTIR spectra: Fourier transform infrared spectra
M1, M2, M4, M7: monomer M1, monomer M2, monomer M4, monomer M7
MA: maleic anhydride
ONL: optical non-linear
P: average laser power
PL: photoluminescence
RA: roughness average
RMS: root mean square
SEM: scanning electron microscopy
TPF: two-photon fluorescence emission
UV-Vis: ultraviolet-visible
*λ*: wavelength
